# Incidence of fractures among children and adolescents in rural and urban communities - analysis based on 9,965 fracture events

**DOI:** 10.1186/2197-1714-1-14

**Published:** 2014-06-02

**Authors:** Erik M Hedström, Ingeborg Waernbaum

**Affiliations:** 1 Division of Surgery and Perioperative Sciences, Umeå University, 901 87 Umeå, Sweden; 2 Department of Statistics, Umeå University, 901 87 Umeå, Sweden

**Keywords:** Child, Fracture, Paediatric, Ecological, Epidemiology

## Abstract

**Background:**

Previous work has explored the significance of residence on injuries. A number of articles reported higher rates of injury in rural as compared to urban settings. This study aimed to evaluate the importance of residency on the occurrence of fractures among children and adolescents within a region in northern Sweden.

**Methods:**

In a population based study with data from an injury surveillance registry at a regional hospital, we have investigated the importance of sex, age and place of residency for the incidence of fractures among children and adolescents 0-19 years of age using a Poisson logistic regression analysis. Data was collected between 1998 and 2011.

**Results:**

The dataset included 9,965 cases. Children and adolescents growing up in the most rural communities appeared to sustain fewer fractures than their peers in an urban municipality, risk ratio 0.81 (0.76-0.86). Further comparisons of fracture rates in the urban and rural municipalities revealed that differences were most pronounced for sports related fractures and activities in school in the second decade of life.

**Conclusion:**

Results indicate that fracture incidence among children and adolescents is affected by place of residency. Differences were associated with activity at injury and therefore we have discussed the possibility that this effect was due to the influence of place on activity patterns.

The results suggest it is of interest to explore how geographic and demographic variables affect the injury pattern further.

**Electronic supplementary material:**

The online version of this article (doi:10.1186/2197-1714-1-14) contains supplementary material, which is available to authorized users.

## Background

Every third child is expected to sustain a fracture before age 17 (Cooper et al. 2004). Previous work has shown a variation in the incidence of fractures with age (Cooper et al. 2004; Landin 1983; Rennie et al. 2007; Hedström et al. 2010). Also, in all epidemiological studies of fractures in children known to us, approximately 60% of all fractures occur in boys (Cooper et al. 2004; Landin 1983; Rennie et al. 2007; Hedström et al. 2010; Lyons et al. 1999).

Previous work has explored the significance of residence on injuries. Several studies investigating urban–rural differences among children and adolescents have reported higher rates in rural settings (Carey et al. 1993; Danseco et al. 2000; Hammig and Weatherley 2003; Owen et al. 2008; Singh et al. 2012). There are also studies that have shown no significant differences (Overpeck et al. 1997; Ni et al. 2002; Coben et al. 2009), and higher rates of injuries among children in urban areas (Gilbride et al. 2006).

In this paper we investigate if living in a rural or urban community influences the incidence of fractures. Sex, and age were included in the explanatory model as potential confounders. A Poisson regression analysis was used.

## Methods

Umeå is situated in northern Sweden. The city itself is predominantly urban whereas the surrounding municipalities; Vännäs, Nordmaling, Robertsfors, Vindeln and Bjurholm are smaller and less densely populated. The population (all ages), population density 0–19 years of age (y.o.a.), and population at risk 0–19 y.o.a. in each municipality are shown in Table 1. Umeå, which is the county capital, has a university and a regional hospital. Health care and social services are two of the main areas of employment for all municipalities. Other principal areas of employment per municipality are shown in Table 1.

**Table 1:** **Demographic variables for the six municipalities within the study**

Municipality	Population all ages*	Population 0–19 years of age*	Child density/km ^2†^	Principal areas of employment (other than health care)**
**Umeå**	112,728	25,790	11.1	Education, Trade, Manufacturing
**Vännäs**	8357	2276	4.3	Manufacturing, Education
**Nordmaling**	7276	1831	1.5	Manufacturing, Trade
**Robertsfors**	6900	1831	1.4	Manufacturing
**Vindeln**	5613	1338	0.5	Manufacturing
**Bjurholm**	2516	587	0.4	Manufacturing, Agriculture

^†^Average population density 0–19 y.o.a. 1998–2011.

*Average annual figure 1998–2011.

**In all municipalities health care and social services employ 19-22% of the work force, the table displays other principal areas of employment within each municipality.

Umeå University Hospital is the only hospital in the area and the emergency department (E.D.) serves as the primary referral point for all patients with known or suspected fractures. The Distance from each municipal centre to the E.D. is as follows; Umeå 2 km, Vännäs 34 km, Vindeln 58 km, Nordmaling 55 km, Robertsfors 59 km, Bjurholm 61 km. Emergency care is free of charge until the age of 19.

Since 1993 all injuries seen at the hospital E.D. are recorded in a database. On arrival patients, or next of kin, are asked to fill in a questionnaire to give information concerning the circumstances of the injury event. This information is fed into the database by employees at the hospital’s Injury Surveillance group who also add to it using the physician’s notes, chart, and ambulance reports etc. We have previously presented an epidemiological overview of fractures in children and adolescents. This paper discussed issues regarding the validity of data (Hedström et al. 2010).

Fractures, in this dataset, were in most cases radiographically confirmed. Rib and nose fractures were in some cases diagnosed only from clinical findings. Patients with fractures were routinely referred to the physician on call from the departments of orthopaedics, hand surgery and ear nose and throat for decisions on treatment and follow up.

The total number of injury events resulting in at least one fracture was 10,292. The municipality of residence was coded in the database but was missing in 327 of these cases (3.2%). These cases were excluded from the regression analysis leaving 9,965 events. For the analysis the municipalities of Nordmaling, Robertsfors, Bjurholm, and Vindeln were considered a unit (NRBV). Vännäs was considered by itself as the demographic profile lay somewhere between Umeå and the other municipalities. Population at risk figures were collected from Statistics Sweden. The average population per year aged 0–19 in Umeå and the five surrounding municipalities 1998–2011 was 33,653. The age variable was divided into four categories 0–4, 5–9, 10–14, 15–19. For subgroup analysis we also made use of information on activity at injury recorded in the database.

### Statistical analysis

For the Poisson family of distributions generalized additive models (GAM) are fitted with a log link function (Hastie and Tibshirani 1990). In GAM-models smoothing terms are allowed giving flexible, nonlinear modelling of selected covariates. The smoothing terms are used for the time trend (Year) and are implemented by a penalized regression spline approach. The response variable is a rate rather than a count, as customary for the Poisson model, hence the population size in the respective age-sex group is included as an offset for each of the models in Table 2. Each variable (age, sex and municipality) was tested for significant dependence in a univariate model and added to the multivariate model according to the strength of dependence. We also tried a model adding an interaction variable for age and sex. This had a negligible effect on the proportion of variability and was therefore left out of the final GAM analysis.

**Table 2:** **Generalized additive models (GAM) for the Poisson family with a log link**

Anova	Residual deviance	Change in deviance	P-value
**1) Null model**	4569.7		-
**2) Year**	4541.7	27.98	<0.0001
**3) Year + Age**	3194.0	1347.74	<0.0001
**4) Year + Age + Sex**	2767.4	426.60	<0.0001
**5) Year + Age + Sex + Area**	2709.4	57.99	<0.0001

The models are evaluated with the response variable being the incidence of fractures. In the model the time trend is fitted with a smooth term and linear coefficients are estimated for the variables sex, age and region.

For the data analysis we use the statistical software R (R Development Core Team 2012). The GAM models are fitted with the functions gam and anova.gam from the package mgcv. Incidences are presented as age and sex adjusted incidence density rates, unless specified, using national figures for Sweden in the year 2000 as the standard population. Subgroups were compared by calculating risk ratios with 95% confidence intervals.

## Results

The incidence of fractures was 223 (219–228)/10^4^ py. The sex specific age adjusted incidence was 264 (258–271)/10^4^ py and 187 (181–193)/10^4^ py, for boys and girls respectively. The incidence per municipality was as follows; Umeå 224 (219–229)/10^4^ py, Vännäs 223 (206–239)/10^4^ py and NRBV 182 (173–192)/10^4^ py.

Each variable was statistically dependent and added according to the strength of the dependence, see Table 2.

Table 3 shows the exp(β), or risk ratio, between subgroups within each variable; year, age, sex, and municipality.

**Table 3:** **Fitted GAM coefficients from model 5**

Anova	Exp(Beta)	95% CI for exp(Beta)	P-value
**Intercept**			<0.0001
**Smooth term (Year)**			<0.0001
Age 5-9*	1.83	1.70-1.96	<0.0001
**Age 10-14**	2.97	2.78-3.17	<0.0001
**Age 15-19**	1.68	1.57-1.80	<0.0001
**Sex (boys)**	1.52	1.46-1.58	<0.0001
**Area (Vännäs)** ^**†**^	1.01	0.93-1.09	0.76
**Area (NRBV)** ^**†**^ **	0.81	0.76-0.86	<0.0001

*With age 0–4 as the reference. ^†^With Umeå as the reference.

**NRBV (Nordmaling, Robertfors, Bjurholm, Vindeln).

(Example of parameter interpretation: Given the time trend, sex and area the estimated rate for children aged 5–9 is 1.83 times that for the younger group aged 0–4) R^2^: 0.81.

The risk ratio between the most rural areas (NRBV) and Umeå was 0.81 (0.76-0.86). The incidence between Umeå and Vännäs didn’t differ significantly. With exclusion of minor fractures; of the ribs, nose, fingers and toes the incidence ratio was still significant between the most rural communities and Umeå/Vännäs 0.85 (0.78-0.91).

Further sub-group analysis was focused on Umeå and the four most rural municipalities. Age and sex specific incidences were compared. These showed a significant difference in those 10–19 y.o.a, most pronounced in boys 10–14 y.o.a. risk ratio 0.72 (0.64-0.86) and girls 15–19 y.o.a. risk ratio 0.74 (0.59-0.89). Further analysis of activity at injury revealed that the observed difference, Umeå-NRBV, was explained mainly by a significant risk ratio for sports related fractures and to a lesser extent activities in school (other than sports). There was no significant difference in the incidence of traffic-related fractures between municipalities.

There were variations over time but no consistent trend towards an increase or decrease in fracture incidence.

## Discussion

The analysis showed a significant relationship between all variables and the incidence of fractures. As expected the greatest incidence was observed in the 10–14 year old age group. Also, as expected there was a predominance of boys who had a 1.52 (1.46-1.58) risk ratio for sustaining a fracture. This is in line with previous results (Cooper et al. 2004; Landin 1983; Rennie et al. 2007; Lyons et al. 1999).

It appeared that living in one of the most rural municipalities had a protective effect, decreasing the risk of sustaining a fracture. Considering the distance from some communities to the hospital we tried to investigate if rates may have been influenced by distance. We excluded minor fractures, which we assumed would be more sensitive to this type of confounding. However, the risk ratio remained significant. Therefore we concluded that the distance to the hospital E.D. did not explain the observed difference in attendance, similar to the results of Lyons et al. (2000).

Municipality, our measure of residence, has several demographic, socioeconomic, and environmental factors nested within it. Which, among these, influenced fracture incidence? The population density for children (which may influence the possibility of gathering two teams for a game of football, or the number of children observed playing on the nearest snow mound) could be one factor. Living in a sparsely populated rural community, with only a few peers living close by, would influence the type of peer interactions and the dynamics of these. The fact that there was little difference between Umeå and Vännäs but significant difference between these and the four most rural communities may indicate that the threshold density lies somewhere between these two groups of communities, i.e. less than four children/km^2^.

Our findings are contrary to several previous comparisons of injury rates among youth in rural and urban settings (Danseco et al. 2000; Hammig and Weatherley 2003; Singh et al. 2012; Mihalicz et al. 2010). These studies have reported higher rates in rural populations for; self-reported medically attended injuries (Danseco et al. 2000), injuries resulting in visits to an emergency department collected from registry data (Owen et al. 2008; Boland et al. 2005), and fatal injuries (Carey et al. 1993; Hammig and Weatherley 2003; Singh et al. 2012; Boland et al. 2005). One study, by Gilbride and colleagues (2006), has reported results pointing in a similar direction to ours. They compared rates for all medically attended injuries retrieved through an administrative registry and found that children and adolescents 0–17 y.o.a. in Alberta, Canada presented higher rates of injuries in urban communities compared to rural communities RR 1.06 (1.05-1.07). This study defined rural communities as those with less than 4000 points of call.

As subgroup analysis showed sports related fractures explained a large proportion of the observed difference. It may indicate that exposure to sports, and the type of sports practiced differed between the municipalities. If this was really the case could not be determined because we had no data on average exposure to sporting activities per child or municipality.

Rose and colleagues (2008) reported that the variable most strongly linked with sports injury was exposure (hours/week). Somewhat contrary to our results they also reported lower rates of sports related injuries in city youth (Calgary and Edmonton) compared to youth from smaller metropolitan and rural areas.

We found no indication of disparities in traffic related fractures. Traffic density would be expected to be less in a rural community; on the other hand children in rural communities may be expected to travel further distances to visit friends and attend school. Previously, injuries sustained by motor vehicle occupants were reported to be more common in rural communities (Carey et al. 1993; Hammig and Weatherley 2003; Boland et al. 2005), whereas pedestrian injuries were more common in urban communities (Hammig and Weatherley 2003; Boland et al. 2005).

Comparisons are generally made difficult by the differences in definition of urban and rural areas, and by the difference in choice of outcome. With regard to the population size and population density all five surrounding municipalities could be considered rural, however we kept Vännäs separate in the analysis as the demographic profile was 'less’ rural than for the other four municipalities. We have tried to provide basic demographic information about each municipality. To our knowledge, the only previous study that presents comparative rates for fractures in relation to urban versus rural residency is by Jiang and colleagues (2007). They reported lower rates when comparing large metropolitan areas, defined as cities with a population greater than one million, with less populated areas. There was no significant difference in fracture rate between “smaller metropolitan areas”, which would correspond in population size to Umeå, and “rural areas”.

### Limitations

We must recognize that our residency variable is a blunt measure of demographic differences. Municipalities are also very heterogeneous with respect to socioeconomic and environmental variables that would ideally also be used when investigating the relationship between area and occurrence of injuries. This was not possible with the material at hand. Studies investigating the relationship between socioeconomic factors and injuries previously have shown relationships, in some cases for unspecified injuries (Owen et al. 2008; Jiang et al. 2007; Reading et al. 1999; Faelker et al. 2000; Xinjun et al. 2008), whereas others have shown a relationship only with certain types of injuries (Gilbride et al. 2006; Dougherty et al. 1990; Reimers and Laflamme 2004; Menon et al. 2008), such as fractures (Lyons et al. 2000) and sports related and recreational injuries (Ni et al. 2002). To reach further conclusions about the factors influencing the observed differences on the municipal level it would be of interest to direct future studies towards comparing smaller, more homogenous areas that are described in greater detail with respect to demographic, but also socioeconomic and environmental variables. Ideally one would also like to collect individual level data for some of these variables together with data on e.g. exposure to sports activities.

Being a one centre study, results and interpretations concerning the differences between rural and urban areas can’t be generalized to other populations.

There were 327 cases with missing data for the residency variable, this could have led to an incorrect estimate of differences. However, had all missing cases come from the four most rural municipalities the geographical risk ratio still would have been significant. No municipal centre lies closer to another hospital. Still, it is possible that families living in the most peripheral parts of the catchment area sought care elsewhere.

A strength of the study is its use of a well-established database. The proportion of unregistered cases has been shown to be stable over time (Hedström et al. 2010). The population at risk was well defined and the hospital’s role as the only referral unit with a radiology department for confirming fractures was also a strength. We have provided age and sex specific rates. This is something that added to the analysis, together with knowledge of the activity at injury, which is lacking in many previous studies.

## Conclusions

We confirmed that age and sex are relevant variables in an explanatory model predicting fracture incidence. We also found that there was a lower rate of fractures in rural and less densely populated municipalities, and that place of residency affected the proportion of variability.

## Consent

No written informed consent was obtained by the patient or patient’s guardian for this particular report. However, the study has been approved by the Ethics Committee of Umeå University.

## Author’s contributions

EMH compiled the description of municipalities, validated the data and performed the subgroup analysis. IW performed the Poisson regression analysis and time trend analysis. EMH and IW planned the study, discussed the analysis and wrote the manuscript. Both authors have approved the final version.

## Authors’ original submitted files for images

Below are the links to the authors’ original submitted files for images. Authors’ original file for figure 1 Authors’ original file for figure 2

## Abbreviations

y.o.a.: Years of age
E.D.: Emergency department
NRBV: The four municipalities Nordmaling, Robertsfors, Bjurholm, and Vindeln.
